# Cost-effectiveness of the Self-Help Plus Intervention for Adult Syrian Refugees Hosted in Turkey

**DOI:** 10.1001/jamanetworkopen.2022.11489

**Published:** 2022-05-10

**Authors:** A-La Park, Tamara Waldmann, Markus Kösters, Federico Tedeschi, Michela Nosè, Giovanni Ostuzzi, Marianna Purgato, Giulia Turrini, Maritta Välimäki, Tella Lantta, Minna Anttila, Johannes Wancata, Fabian Friedrich, Ceren Acartürk, Zeynep İlkkursun, Ersin Uygun, Sevde Eskici, Pim Cuijpers, Marit Sijbrandij, Ross G. White, Mariana Popa, Kenneth Carswell, Teresa Au, Reinhold Kilian, Corrado Barbui

**Affiliations:** 1 Department of Psychiatry and Psychotherapy II, University of Ulm and BKH Günzburg, Ulm, Germany; 2 Care Policy and Evaluation Centre, Department of Health Policy, London School of Economics and Political Science, London, United Kingdom; 3 WHO Collaborating Centre for Research and Training in Mental Health and Service Evaluation, Section of Psychiatry, Department of Neuroscience, Biomedicine and Movement Sciences, University of Verona, Verona, Italy; 4 Department of Nursing Science, University of Turku, Finland; 5 School of Nursing, Central South University, Changsha Hunan, China; 6 Clinical Division of Social Psychiatry, Department of Psychiatry and Psychotherapy, Medical University of Vienna, Vienna Austria; 7 Department of Psychology, Koc University, Sariyer, Istanbul,Turkey; 8 Trauma and Disaster Mental Health, Istanbul Bilgi University, Eyüpsultan/Istanbul, Turkey; 9 Department of Psychology, Istanbul Koc University, Sariyer, Istanbul, Turkey; 10 Department of Clinical, Neuro- and Developmental Psychology, Amsterdam Public Health Research Institute, Vrije Universiteit Amsterdam, Amsterdam, the Netherlands; 11 Institute of Population Health, University of Liverpool, Liverpool, United Kingdom; 12 School of Psychology, Queen’s University Belfast, Belfast, United Kingdom; 13 Department of Mental Health and Substance Use, World Health Organization, Geneva, Switzerland

## Abstract

**Importance:**

The cost-effectiveness of the Self-Help Plus (SH+) program, a group-based, guided, self-help psychological intervention developed by the World Health Organization for people affected by adversity, is unclear.

**Objective:**

To investigate the cost-utility of providing the SH+ intervention combined with enhanced usual care vs enhanced usual care alone for Syrian refugees or asylum seekers hosted in Turkey.

**Design, Setting, and Participants:**

This economic evaluation was performed as a prespecified part of an assessor-blinded randomized clinical trial conducted between October 1, 2018, and November 30, 2019, with 6-month follow-up. A total of 627 adults with psychological distress but no diagnosed psychiatric disorder were randomly assigned to the intervention group or the enhanced usual care group.

**Interventions:**

The SH+ program was a 5-session (2 hours each), group-based, stress management course in which participants learned self-help skills for managing stress by listening to audio sessions. The SH+ sessions were facilitated by briefly trained, nonspecialist individuals, and an illustrated book was provided to group members. Th intervention group received the SH+ intervention plus enhanced usual care; the control group received only enhanced usual care from the local health care system. Enhanced usual care included access to free health care services provided by primary and secondary institutions plus details on nongovernmental organizations and freely available mental health services, social services, and community networks for people under temporary protection of Turkey and refugees.

**Main Outcomes and Measures:**

The primary outcome measure was incremental cost per quality-adjusted life-year (QALY) gained from the perspective of the Turkish health care system. An intention-to-treat analysis was used including all participants who were randomized and for whom baseline data on costs and QALYs were available. Data were analyzed September 30, 2020, to July 30, 2021.

**Results:**

Of 627 participants (mean [SD] age, 31.3 [9.0] years; 393 [62.9%] women), 313 were included in the analysis for the SH+ group and 314 in the analysis for the enhanced usual care group. An incremental cost-utility ratio estimate of T£6068 ($1147) per QALY gained was found when the SH+ intervention was provided to groups of 10 Syrian refugees. At a willingness to pay per QALY gained of T£14 831 ($2802), the SH+ intervention had a 97.5% chance of being cost-effective compared with enhanced usual care alone.

**Conclusions and Relevance:**

This economic evaluation suggests that implementation of the SH+ intervention compared with enhanced usual care alone for adult Syrian refugees or asylum seekers hosted in Turkey is cost-effective from the perspective of the Turkish health care system when both international and country-specific willingness-to-pay thresholds were applied.

## Key Points

**Question:**

Is the addition of the World Health Organization’s Self-Help Plus program—a group-based, guided, self-help psychological intervention—to enhanced usual care cost-effective compared with enhanced usual care alone for adult Syrian refugees or asylum seekers hosted in Turkey?

**Findings:**

In this economic evaluation of 627 refugees or asylum seekers hosted in Turkey, the intervention group had a significantly better quality of life compared with the control group, at a cost of 6068 Turkish lira ($1147) per quality-adjusted life-year gained. Taking into account the stochastic uncertainty, the program had a 97.5% chance of being cost-effective.

**Meaning:**

These findings suggest that Self-Help Plus is cost-effective as an intervention to prevent mental disorders in conflict-exposed refugees hosted in Turkey.

## Teaser

This economic evaluation assesses whether providing the World Health Organization’s Self-Help Plus intervention combined with enhanced usual care is cost-effective compared with enhanced usual care alone for conflict-exposed Syrian refugees or asylum seekers hosted in Turkey.

## Introduction

People who are seeking shelter as refugees or asylum seekers from violent conflicts or political persecution show increased prevalence of mental disorders, particularly posttraumatic stress disorder, anxiety and depression, compared with the general population in the host countries.^1,2,3^ Although governments of most host countries, often in collaboration with national and international nongovernmental organizations, provide at least emergency care for refugees or asylum seekers, to date the availability of evidence-based mental health and psychosocial support programs for this group is insufficient.^3,4^

Turkey currently hosts 3.6 million Syrian refugees or asylum seekers, which is more than 50% of the Syrian refugees or asylum seekers pursuing shelter from the civil war in Syria.^5^ The prevalence of common mental disorders, including depression, anxiety and posttraumatic stress disorder, among Syrian refugees or asylum seekers hosted in Turkey is as high as among other refugees or asylum seeker populations who have fled from civil war regions.^6,7,8^ Even though Syrian refugees or asylum seekers in Turkey have access to the public mental health care system, a recent investigation of mental health service use among adult Syrian refugees or asylum seekers living in Istanbul revealed that 90% of respondents who screened positive for common mental health disorders did not use any mental health care services.^9,10,11^ Given this large treatment gap even among people who are likely to have a mental disorder, there is a high probability that subclinical mental health problems remain largely undetected and a risk of increasing symptom severity if problems are not identified and addressed.^12^ Therefore, preventative interventions that strengthen the coping capacity of people who are at increased risk of developing a mental disorder are considered a useful supplement to professional mental health care.^13^

Self-Help Plus (SH+) is a group-based, guided, self-help psychological intervention developed by the World Health Organization (WHO) to increase the stress-management capacity of adults exposed to adversity, reduce psychological distress, and prevent the onset of mental disorders.^14^ The SH+ intervention prevented the onset of mental disorders among adult Syrian refugees in Turkey at a 6-month follow-up assessment.^13,15,16^

Although SH+ is a low-cost intervention that has been shown to be effective, funders and health policymakers require information on the cost-effectiveness of SH+ that can be compared with that of other, competing health care interventions for this highly vulnerable target group. Incremental cost-utility analysis provides such information by revealing the maximum willingness-to-pay (WTP) level needed to gain an additional life-year in complete health by means of the evaluated intervention.^17^

This economic evaluation presents the results of a cost-utility analysis for the SH+ intervention in addition to enhanced usual care compared with enhanced usual care alone for adult Syrian refugees or asylum seekers in Turkey, covering a 6-month period. Our economic evidence may help funders and health policymakers make informed decisions about the allocation of scarce resources.

## Methods

The data for this health economic evaluation have been gathered as part of the RE-DEFINE project (Implementation of Self Help Plus in Adult Syrian Refugees in Turkey), which comprised 2 randomized controlled trials for investigating the efficacy of the SH+ intervention in 5 European countries (Austria, Finland, Germany, Italy, and UK) and Turkey.^13^ The design and the main results of the RE-DEFINE project are published elsewhere.^13,18^ In the present article, we present the health economic evaluation of the Turkish trial. This study follows the Consolidated Health Economic Evaluation Reporting Standards (CHEERS) reporting guideline.^19^ The study was approved by the WHO Research Ethics Review Committee and the ethics committees of Istanbul Sehir University and Koc University. No participant received compensation or incentive for participating in this study. Data were analyzed September 30, 2020, to July 30, 2021.

We conducted an incremental cost-utility analysis from the perspective of the Turkish health and social care system. Because the time horizon of the study was 12 months, we did not discount costs and results. Inclusion criteria were (1) age of 18 years or older, (2) ability to speak and understand Levantine Arabic, (3) status as Syrians under temporary protection (4) positive result (score ≥3) on the 12-item General Health Questionnaire (range, 3-12, with 3 indicating mild psychological distress and 12 indicating severe psychological distress), and (5) ability to provide oral and written informed consent for participation. Exclusion criteria were having (1) a diagnosis of any psychiatric disorder according to the Mini-International Neuropsychiatric Interview, (2) an acute medical condition that inhibited study participation, (3) an imminent risk of suicide, or (4) clinical evidence that decision-making capacity was impaired.

### The SH+ Course

The SH+ course is a 5-session, group-based, stress management course developed by WHO for people affected by adversity.^20^ It has 2 main components: a prerecorded audio course^14^ and an illustrated book.^14^ During five 2-hour sessions, participants learned self-help skills for managing stress by listening to the audio course in groups of up to approximately 30 people. Briefly trained, nonspecialist facilitators reviewed the skills introduced by the audio course and read discussion questions to the group. The stress management skills were further reinforced by the illustrated book, which participants could use between sessions to review the core skills taught in the audio course.

### Enhanced Usual Care

Similar to all refugees under temporary protection in Turkey, participants in both groups received enhanced usual care and had access to free health care services provided by primary and secondary institutions (eg, health stations, health centers, maternal-infant care, family planning centers, tuberculosis dispensaries, and state hospitals). There are also approximately 200 Migrant Health Centers, which are similar to family practice (primary health care) services in Turkey. The control group received enhanced usual care alone.

In addition, participants received baseline and follow-up assessments at prespecified times, contact details for nongovernmental organizations, and information about freely available mental health services, social services, and community networks providing support to people under temporary protection of Turkey and refugees.

### Measures

To assess health service use and costs, we used the European version of the Client Socio-Demographic and Service Receipt Inventory.^21^ To adapt the inventory for refugees or asylum seekers, we sent the original version of the inventory to participating study centers with a request for comments regarding the spectrum of health and social care services available for refugees or asylum seekers in Turkey. On the basis of the received comments, a Turkish version of the inventory for refugees or asylum seekers was developed and returned to the participating centers with a request for additional comments and for final approval. Health and social services use was retrospectively assessed at baseline and at the 6-month follow-up by trained research workers. Because no Syrian version of the EuroQol 5-Dimensions Scale was available, the Arabic version for Saudi Arabia was used for participants. The EuroQol 5-Dimensions Scale was assessed at baseline and at the 6-month follow-up.

### Cost Analysis

Comprehensive costs of health and social care services were estimated by unit costs provided by experts from the participating Turkish study (C.A., Z.İ., and E.U.). Owing to the lack of 12-month follow-up data, annual costs were estimated by summing 6-month costs at baseline with costs at the 6- month follow-up. For the intention-to-treat analysis, we imputed missing cost data at follow-up by carrying forward baseline cost information.

Total intervention costs per group were estimated by summing the costs of facilitator training, facilitator allowances, and overhead. Intervention costs per participant were estimated by dividing the total per group costs by the mean number of participants in the initial SH+ sessions. Costs are reported in Turkish lira (T£, 2019 price) and US dollars (to convert to US $, T£ values were multiplied by 0.18898, which was the mean exchange rate in 2019).

### Statistical Analysis

The incremental cost-utility analysis^22^ was conducted from the perspective of the Turkish health and social care system. Quality-adjusted life-years (QALYs) were generated using the UK value set because neither a value set for refugee populations nor country specific value sets for the Turkish or the Syrian populations were available.^23^ Cost differences and QALY differences between the 2 groups were estimated using generalized linear regression models with a gamma family distribution and a log link. The skewed distribution of the cost data was taken into account by estimating robust standard errors using the Huber-White sandwich estimator.^24^

Incremental cost-utility ratios (ICURs) were estimated indicating the cost of 1 additional QALY gained by providing SH+ in addition to enhanced usual care, compared with enhanced usual care alone.

The stochastic uncertainty of the ICUR was estimated by nonparametric bootstrapping with 10 000 replications.^22,25^ Two-sided 95% CIs for the ICUR were estimated using the percentile method.^22,25^ Cost-effectiveness acceptability curves and net monetary benefits were estimated for a maximum WTP range between T£0 and T£125 000 ($0-$23 623).^22^ Sensitivity analyses were conducted to model different intervention costs with a group size of 5 as the most expensive scenario, a group size of 10 as the most likely scenario, and a group size of 25 as the least expensive scenario. Additional information on applied cost-utility methods is provided in the eMethods and eFigure in the Supplement. Analyses were conducted using Stata, version 17 (StataCorp LLC). A 2-sided *P* < .05 was considered statistically significant.

## Results

A total of 642 individuals were randomly allocated to the intervention group (SH+, 322) or to the control group (enhanced usual care, 320) (eTable 1 in the Supplement). However, owing to missing cost data at both assessment times, 15 participants who were randomized (9 to SH+ and 6 to enhanced usual care) were excluded from the analysis. No differences in sample characteristics were obtained between included and excluded cases (eTable 2 in the Supplement). Thus, the study sample for our economic analyses comprised 627 participants, with 313 in the SH+ group and 314 in the enhanced usual care group. The mean (SD) age of the participants was 31.3 (9.0) years, 393 (62.7%) were women, 234 (37.3%) were men, 505 participants (80.5%) reported having a partner, and the mean (SD) duration of education was 9.1 (3.7) years.

As indicated in Table 1, only 82 of the study participants (13.1%) reported any use of physical or mental health care services during the previous 12 months. The most frequently used services were outpatient treatment (42 participants [6.7%]) and inpatient treatment (30 participants [4.8%]). The use of any medication was reported by 20 participants (3.1%). Owing to the small number of participants who used any health service, the mean (SD) annual health care costs were low (T£20.51 [T£111.30]; $3.88 [$21.03]) per participant. No statistically significant differences in health services use were identified between the 2 groups.

**Table 1.** Health Service Use and Mean Annual Costs of Health Service Use by Study Group

Health service	Total (N = 627)		Enhanced usual care (n = 314)		SH+ (n = 313)		Cost for SH+ minus enhanced usual care (*P* value for difference)^b^
	Participants, No. (%)	Mean cost per participant, (SD), T£ ($)^a^	Participants, No. (%)	Mean (SD) cost per participant, (SD), T£ ($)^a^	Participants, No. (%)	Mean (SD) cost per participant, (SD), T£ ($)^a^	
Inpatient treatment	30 (4.8)	3.46 (20.9) (0.65 [3.94])	14 (4.5)	3.31 (22.30) (0.63 [92.51])	16 (5.1)	3.60 (19.40) (0.68 [3.67])	0.29 (.86)
Outpatient treatment	42 (6.7)	9.48 (55.2) (1.97 [10.58])	20 (6.4)	7.72 (41.40) (1.46 [7.83])	22 (7.0)	11.26 (67.50) (2.13 [12.76])	3.54 (.43)
Community mental health care	7 (1.1)	2.92 (35.40) (0.55 [6.70])	5 (1.6)	3.06 (34.80) (0.58[6.57])	2 (0.6)	2.79 (36.2) (0.53 [6.84])	–0.27 (.92)
Primary care center	8 (1.3)	4.26 (58.70) (0.81 [11.09])	3 (1.0)	5.96 (78.80) (1.13 [14.90])	5 (1.6)	2.56 (25.90) (0.48 [4.89])	–3.40 (.47)
Medications	20 (3.1)	0.38 (2.80) (0.07 [0.53])	5 (1.6)	0.27 (2.70) (0.05 [0.51])	15 (4.8)	0.49 (2.90) (0.09 [0.55])	0.23 (.31)
Any service use and total costs except SH+	82 (13.1)	20.51 (111.30) (3.88 [21.03])	36 (11.5)	20.32 (111.90) (3.84 [21.15])	46 (14.7)	20.70 (110.9) (3.91 [20.96])	0.38 (.97)
SH+ intervention, group size of 10 persons	313 (49.9)	193.80 (0.00) (36.62 [0.00])	0	0	313 (100)	193.80 (0.00) (36.62 [0.00])	193.80
Total cost including SH+ intervention	627 (100)	146.54 (255.51) (27.69 [48.29])	36 (11.5)	20.32 (111.89) (3.84 [21.14])	313 (100)	214.49 (110.89) (40.53 [48.29])	194.18 (<.001)

Abbreviations: SH+, Self-Help Plus; T£, Turkish lira.

^a^ To convert to US dollars, Turkish lira values are multiplied by 0.18898, the mean exchange rate in 2019.

^b^ Cost difference SH+ minus enhanced usual care was tested by generalized linear regression models with log link and gamma family distribution.

The total costs for training the SH+ facilitators, the allowances for the facilitators, and the overhead costs (room rent and traveling costs for facilitators) amounted to T£1938 ($366). The study participants assigned to the intervention group were allocated to 20 groups with 5 group sessions each. The mean (SD) number of participants was 4 (4). The mean (SD) number of participants at each group session was 10 (2). On the basis of these numbers, we estimated the cost per SH+ participant to receive a course of SH+ for a group size of 5 was T£388 ($73); for a group size of 10, T£194 ($37); and for a group size of 25, T£78 ($15) (Table 2).

**Table 2.** Sensitivity Analysis for the Cost Utility of the SH+ Program by Group Size

SH+ group size, No.	SH+ costs per participant, T£ ($)^a^	ICUR point estimate, T£ ($)^a^	ICUR 95% CI	
			Lower limit, T£ ($)^a^	Upper limit, T£ ($)^a^
5	388 (73)	12 516 (2365)	7698 (1455)	30 133 (5695)
10	194 (37)	6068 (1147)	3829 (724)	14 831 (2802)
25	78 (15)	2434 (460)	1432 (271)	6308 (895)

Abbreviations: ICUR, Incremental Cost Utility Ratio; SH+, Self-Help Plus; T£, Turkish lira.

^a^ To convert to US dollars, Turkish lira values are multiplied by 0.18898, the mean exchange rate in 2019.

Including the cost of the SH+ intervention, for a group size of 10 participants, the SH+ group incurred mean (SD) annual costs of T£215 (T£111) ($41 [$21]) compared with the mean (SD) of T£20 (T£112) ($4 [$21]) incurred by participants in the enhanced usual care group. This resulted in a mean (SE) annual cost difference of T£194 (T£9l) ($37 [$2]), which was statistically significant (*P* ≤ .001).

The mean (SD) QALY value was 0.42 (0.11) for participants in the SH+ group and 0.39 (0.13) for participants in the enhanced usual care group. The mean (SE) QALY difference of 0.032 (0.009) was statistically significant (*P* = .001). The differences in costs and QALYs resulted in an ICUR point estimate of T£6068 ($1147), indicating that from the perspective of the Turkish health and social care system, the gain of 1 additional QALY by providing SH+ for a mean group size of 10 in addition to enhanced usual care for Syrian refugees or asylum seekers was associated with an additional cost of T£6068 ($1147).

**Figure 1.**
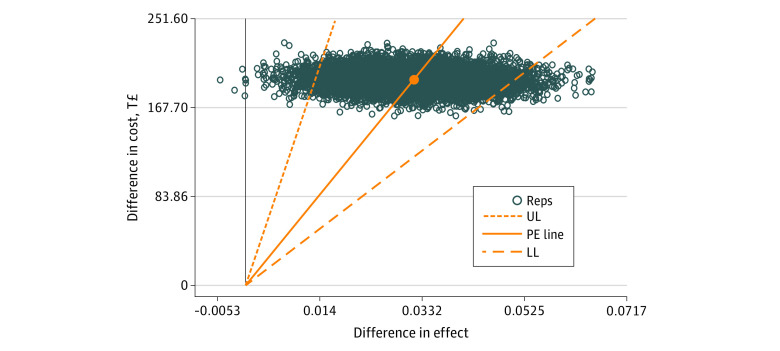
Stochastic Uncertainty Based on Nonparametric Bootstrapping With 10 000 Replications and 95% CI of the Incremental Cost-Utility Ratio (ICUR) for Provision of the Self-Help Plus Program for Syrian Refugees in Turkey The 95% CI estimated for the ICUR represents the fraction of 95% of the bootstrap samples defined by cutting the highest 2.5% and the lowest 2.5% of the ICUR values simulated by the 10 000 bootstrap samples. Orange data point represents the ICUR point estimate; Reps, bootstrap replications of the ICUR; LL, lower limit of the 95% CI; UL, upper limit of the 95% CI; and T£, Turkish lira. PE (point estimate) line represents the slope of the cost-utility point estimator; vertical line, cost difference between treatment alternatives; horizontal line at 0, quality-adjusted life-year difference between treatment alternatives.

**Figure 2.**
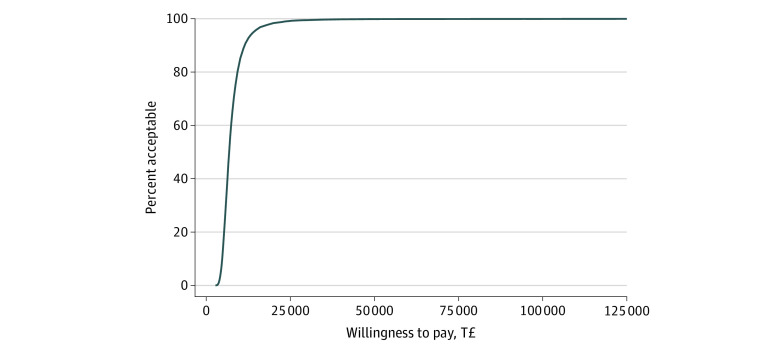
Cost-effectiveness Acceptability Curve (CEAC) of the Cost-Utility for Providing the Self-Help Plus Program for Syrian Refugees in Turkey On the horizontal line at 0, the CEAC shows the potential values for the maximum willingness to pay (MWTP) in increasing order, and the vertical axis shows the percentages of the estimated incremental cost-utility ratio values that are located below the MWTP curve. Similar to the statistical CI, the CEAC indicates at which MWTP a particular percentage of the estimated incremental cost-utility ratio falls below the MWTP curve. A percentage of acceptance of 95% is equivalent to a 1-sided statistical significance of 2.5%. T£ represents Turkish lira.

**Figure 3.**
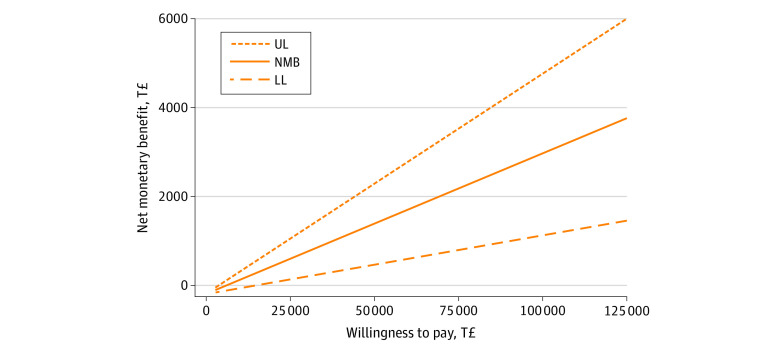
Net Monetary Benefit (NMB) and 95% CI for Providing the Self-Help Plus Intervention vs Enhanced Usual Care for Syrian Refugees in Turkey at the Willingness-to-Pay (WTP) Range of Turkish Lira (T£)0 to T£125 000 (US $0-$23 623) The NMB regression curve represents the monetary gain that the decision-maker may expect from implementing the intervention along a defined range of maximum WTP values between T£0 and T£125 000. A positive NMB may be expected from the maximum WTP value where the lower limit (LL) of the 95% CI of the NMB regression curve passes the x-axis representing the maximum WTP. UL represents upper limit of the 95% CI.

Estimation of the stochastic uncertainty (Figure 1) showed that the ICUR varied in most parts within the upper-right quadrant of the cost-effectiveness plane, indicating that the SH+ intervention was associated with better outcomes and higher costs than enhanced usual care only. The 95% CI of the ICUR for providing SH+ with a mean group size of 10 ranged between a lower limit of T£3829 ($724) and an upper limit of T£14 831($2802). Thus, with a probability of 95%, the SH+ intervention was cost-effective compared with enhanced usual care alone at a WTP level higher than T£14 831 ($2802). This result is supported by the cost-effectiveness acceptability curve presented in Figure 2. This curve indicated a high chance for the SH+ being cost-effective with 97.5% at a WTP level of T£14 831 ($2802) up to T£125 000 ($23 623). These values represented a 2-sided significance level of *P* = .05 for the cost-effectiveness of the SH+ intervention compared with enhanced usual care. The net monetary benefit curve presented in Figure 3 indicated that for a WTP level of T£14 831 ($2802) up to a WTP level of T£125 000 ($23 623), the implementation of the SH+ intervention was associated with a significant net monetary benefit linearly increasing from T£250 ($47) at WTP T£14 831 ($2802) up to T£40 000 ($7559) at WTP T£125 000 ($23 623).

### Sensitivity Analysis

As shown in Table 2, per capita costs of the SH+ intervention varied from T£78 ($15) for the least expensive scenario with a group size of 25 to T£388 ($73) for the most expensive scenario with a group size of 5. The resulting ICUR point estimates varied between T£2434 ($460) per QALY for the least expensive scenario and T£12 516 ($2365) per QALY for the most expensive scenario. Taking into account the ICUR variance resulted in upper limits of the 95% CI of the ICUR between T£6308 ($895) per QALY for the least expensive scenario and T£30 133 ($5695) per QALY for the most expensive scenario.

## Discussion

To the best of our knowledge, this economic evaluation is the first study to report health economic outcomes of a low-intensity, guided, self-help intervention for preventing mental disorders among refugees or asylum seekers in Turkey. As indicated by our results, only a small number of participants used any type of medical or psychiatric service during the 12-month period of assessment. This is in line with previous studies, suggesting that preventive mental health care services are rarely used by refugee populations.^9,26^

The mean annual health care costs of T£20.51 per participant fell well below the per capita health care expenditures in Turkey, which amounted to T£2434 in 2019.^27^ This may suggest either that usual health care services were not sufficiently accessible to these study participants or that the study participants were reluctant to use available services.

Although the absolute per capita costs of SH+ were low even under the assumption of the most expensive scenario, the provision of the intervention in addition to enhanced usual care would increase the mean annual per capita health care costs of the target population significantly even in the best case scenario. The point estimate of the ICUR indicated that the gain of 1 life-year in full health by providing the SH+ intervention for Syrian refugees or asylum seekers compared with enhanced usual care alone costs a mean of T£6068 ($1147). Taking into account the stochastic uncertainty of the ICUR, a WTP of T£14 831 would be needed for the SH+ intervention to be estimated as cost-effective in comparison with enhanced usual care with a probability of 95%.

In the absence of a national guideline for the application of the acceptable maximum WT*P* values, the suggestion of the WHO Choosing Interventions That Are Cost-Effective (WHO-CHOICE) group for country-specific thresholds based on per capita gross domestic product are widely used for the interpretation of cost-utility analysis results.^28,29^ According to the WHO-CHOICE suggestion, an ICUR up to 3 times the annual gross domestic product per capita may be considered good value for money for a health care intervention.^28^ In response to the criticism about a lack of justification for using the gross domestic product per capita approach, there has been a recent development in WTP thresholds for economic evaluations using county-specific opportunity costs.^30^ For Turkey, a study by Woods and colleagues^30^ estimated threshold values ranging from $2950 to $6861, which would be within the WTP range even in the most expensive scenario in our study, assuming the smallest group size of 5 participants per group. Moreover, given the gross domestic product per capita in Turkey (T£51 834; $9127) in 2019, the provision of the SH+ program plus enhanced usual care for Syrian refugees or asylum seekers in the most expensive scenario is still considered highly cost-effective compared with enhanced usual care alone at the maximum WTP of T£30 133 ($4276) from the perspective of the Turkish health care system, regardless of different approaches to thresholds applied.

### Limitations

The assessment of service use based on self-report may be subject to recall bias and participants’ reluctance to report the use of particular treatments or services. This may be the case especially for reporting the use of mental health–related services owing to the stigma associated with mental disorders, which have been linked with high levels of shame in Syrian refugee populations.^31^ Using combined data at baseline and 6-month follow-up to estimate annual costs in addition to last observation carried forward imputation for missing 6-month follow-up cost data had the disadvantage that the potential effects of the intervention on the decrease or increase in health care costs would be disregarded in our analysis. However, repeated analyses with multiple imputation of cost data and with 6-month follow-up costs alone did not change the results (eTable 3 in the Supplement). Application of the United Kingdom utility value sets for deriving QALYs may have resulted in biased assessments as a result of cultural differences or adverse living conditions of the target population.^23^ Owing to the large mental health treatment gap faced by the study population,^9^ any form of extra attention and recognition may have positive effects independent of its specific content, limiting the conclusiveness of our findings.

## Conclusions

The results of this economic evaluation suggest that providing the SH+ intervention—a low-intensity, guided, self-help intervention for preventing mental disorders—for Syrian refugees or asylum seekers in Turkey with psychological distress but without a formal psychiatric diagnosis may increase the quality of life of this population at additional costs far below the internationally accepted thresholds for cost-effectiveness.

## Notes

Supplement. **eTable 1.** Sample Characteristics **eTable 2.** Sample Characteristics Excluded Cases **eTable 3.** Sensitivity Analysis ICUA Results Depending on Data Set and Missing Value Imputation **eMethods.** Additional Information About the Applied Health Economic Methods **eFigure.** Cost-effectiveness Plane
eReferences
