# Supplementary material for: Cost-effectiveness of the Self-Help Plus Intervention for Adult Syrian Refugees Hosted in Turkey
Source: JAMA Netw Open. 2022 May 10;5(5):e2211489. doi: 10.1001/jamanetworkopen.2022.11489 (PMC9092202; doi:10.1001/jamanetworkopen.2022.11489)
Supplement: Supplement. — eTable 1. Sample Characteristics eTable 2. Sample Characteristics Excluded Cases eTable 3. Sensitivity Analysis ICUA Results Depending on Data Set and Missing Value Imputation eMethods. Additional Information About the Applied Health Economic Methods eFigure. Cost-effectiveness Plane eReferences [file jamanetwopen-e2211489-s001.pdf]

Supplementary Online Content

Park AL, Waldmann T, Kösters M, et al. Cost-effectiveness of the Self-Help Plus intervention for adult Syrian refugees hosted in Turkey. *JAMA Netw Open*. 2022;5(5):e2211489. doi:10.1001/jamanetworkopen.2022.11489

eTable 1. Sample Characteristics

eTable 2. Sample Characteristics Excluded Cases

eTable 3. Sensitivity Analysis ICUA Results Depending on Data Set and Missing Value Imputation

eMethods. Additional Information About the Applied Health Economic Methods

eFigure. Cost-effectiveness Plane

eReferences.

This supplementary material has been provided by the authors to give readers additional information about their work.

© 2022 Park AL et al. *JAMA Network Open*.

eTable 1. Sample Characteristics

	Total<br>N = 627	ETAU<br>N = 314	SH+<br>N = 313	p<br>diff
Female n (%)	393 (62.9)	193 (61.5)	200 (63.9)	0.529
Living with partner n (%)	505 (80.5)	255 (81.2)	250 (79.9)	0.672
Age m (SD)	31.3 (9,0)	31.5 (9.0)	31.1 (8.9)	0.540
Years of education m (SD)	9.1 (3.7)	9.1 (3.7)	9.0 (3.7)	0.640

© 2022 Park AL et al. *JAMA Network Open*.

eTable 2. Sample Characteristics Excluded Cases

	Total<br>N = 642	Sample<br>N = 627	Excluded<br>N = 15	p<br>diff
Female n (%)	404 (62.3)	293 (62.9)	11 (73.3)	0.399
Living with partner n (%)	518 (80.7)	505 (80.5)	13 (86.7)	0.553
Age m (SD)	31.5 (9,0)	31.3 (8.9)	37.9 (11.5)	0.005
Years of education m (SD)	9.0 (3.7)	9.1 (3.7)	7.2 (3.8)	0.065

© 2022 Park AL et al. *JAMA Network Open*.

eTable 3. Sensitivity Analysis ICUA Results Depending on Data Set and Missing Value Imputation

Data set<br>Missing value Imputation method	ICUR* point estimate (TRY)	ICUR 95% CI lower limit (TRY)	ICUR 95% CI upper limit (TRY)
Baseline + 6 month follow-up<br>LOCF	6.086	3,829	14,831
6 month-follow-up<br>LOCF	6.158	3,890	15.217
Baseline + 6 month follow-up<br>MI 20 samples	6.066	3.454	23.132
6 month follow-up<br>MI 20 samples	6.219	3.564	23.747

LOCF = Last observation carried forward; MI=Multiple imputation

© 2022 Park AL et al. *JAMA Network Open*.

eMethods. Additional Information About the Applied Health Economic Methods

Estimation of quality adjusted life years by means of the EuroQol EQ-5D questionnaire:

The QALY estimates life years in complete health. This is estimated by multiplying the utility value corresponding to the participants' health state with the time lived in this health state. The utility value is ranging between 0 for the worst possible state of health (which means close to death) and 1 for the best possible state of health (which means full health). The time lived in a particular health state is defined by the questionnaire and depends on the frequency of measures. In our study a six-month time frame was used which means that the participant was asked to assess his or her health state for the last six months. To transform the measure into an annual health state, the utility value for the self-reported health state is multiplied by the fraction of the 12 months period which it represents. That means a utility value for a six-month period is multiplied by 0.5 to represent the QALY for a 12-month period. In case of repeated measures, the fractions of the estimated utility values will be summed up over the 12-month period [1].

Statistical analysis of incremental costs utility

The task of health economics is to provide necessary information for an optimal allocation of health care resources [1]. The main tools of health economic evaluation are the incremental cost-effectiveness analysis (ICEA) or the incremental cost-utility analysis (ICUA). While ICEA considers the additional (incremental) costs of a medical intervention in relations to its additional (incremental) effect on health, ICUA regards incremental costs in relation to the incremental utility for the patient. Using the concept of utility instead of the sole health effect in economic evaluation is considered as more appropriate due to the assumption that the subjective utility of health varies between individuals depending on their personal preferences. The concept of utility is of particular importance when life prolonging treatment may have considerable negative effects on quality of life (QOL) as it is the case in breast cancer treatment. Utility indicators such as the quality adjusted life year (QALY) are created by the combination of saved life years with preference based health related quality of life (HRQOL) measures. In contrast to general HRQOL measures preference based HRQOL measures will be obtained by assessing the values of several health states on a continuum between optimal health and the poorest imaginable health state or death by techniques such as the visual analogue scale (VAS) method, time trade off (TTO), or standard gamble (SG). The outcome of a cost-utility analysis is the incremental cost-utility ratio (ICUR).

© 2022 Park AL et al. *JAMA Network Open*.

$$ICUR = \frac{Cost\ Intervention\ A\ \text{-}\ Cost\ Intervention\ B}{Utility\ Intervention\ A\ \text{-}\ Utility\ Intervention\ B} = \frac{\Delta\ Cost}{\Delta\ Utility}$$

The ICUR is defined as the ratio between the cost differences and the utility differences of two alternative treatments or of a treatment in comparison to no treatment. The interpretation of the ICUR depends on its position on the cost-effectiveness plane (CEP).

© 2022 Park AL et al. *JAMA Network Open*.

eFigure 1. Cost-effectiveness Plane

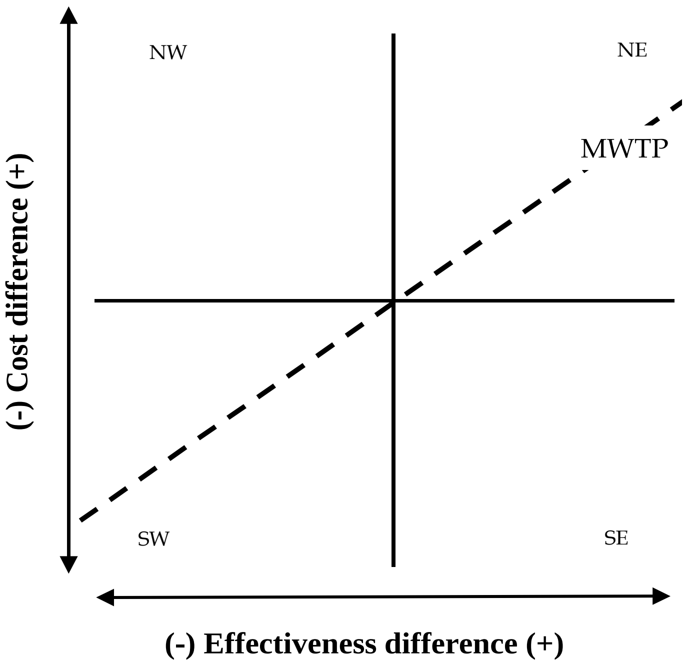

© 2022 Park AL et al. *JAMA Network Open*.

On its vertical axis the CEP (eFigure) shows the cost differences and on its horizontal axis the effectiveness or utility differences between the interventions which should be compared. If the ICUR is located at the upper left (north west) quadrant of the CEP the ICUR has a negative value indicating that the alternative intervention is more expensive and less effective than the conventional or the non-intervention. If the ICUR is located at the upper right (north east) quadrant of the CEP the positive value indicates that the alternative intervention is more effective but also more expensive than the conventional or the non-intervention. If the ICUR is located at the lower left (south west) quadrant of the CEP the positive value indicates that the alternative intervention is less expensive but also less effective than the conventional or the non-intervention. If the ICUR is located at the lower right quadrant of the CEP the negative value indicates that the alternative intervention is less expensive and more effective than the conventional or the non-intervention. Obviously, a negative ICUR provides a much more clear decision basis than a positive one. If the ICUR is located at the upper left quadrant, the conventional or the non-intervention is more efficient than the alternative intervention and if the ICUR is located at the lower right, the alternative intervention is more efficient. Unfortunately, the ICUR mostly falls into the upper right quadrant. In this case an additional criterion is needed to decide whether an alternative treatment is efficient or not. This additional criterion is the maximal amount of money which one is willing to pay for an increase of the effect or the utility by one unit and is usually called maximum willingness to pay (MWTP).
The MWTP can be projected as a growth curve into the CEP and an alternative intervention will be assessed as efficient if the ICUR is located below this curve.
Estimation of the stochastic uncertainty of the ICUR is complicated by the fact that a ratio of differences between the study groups cannot be computed at the individual level but only at the level of the whole study sample. As a solution for this problem, bootstrapping techniques will be used to simulate the true sampling distribution of the ICUR [2, 3]. A further complication results from the fact that the ICUR ($\Delta C/\Delta E$) distribution is not defined for $\Delta E=0$. As a consequence the confidence interval for the ICUR cannot be estimated based on parametric assumptions about the mean and the SD [2]. The 95% confidence interval estimated for the ICUR represents the fraction of 95 % of the bootstrap samples defined by cutting the highest 2.5 % and the lowest 2.5 % of the ICUR values simulated by the bootstrap resampling[1].

The cost-effectiveness acceptability curve (CEAC) is the common technique to visualize this criterion [1, 2]. At the horizontal axis the CEAC shows potential values for MWTO in an increasing order, the vertical axis shows the percentages of the estimated ICUR values which are located below the MWTP curve. Similar as the statistical confidence interval the CEAC indicates at which MWTP a particular percentage (eg 90%) of the estimated ICUR fall below the MWTP curve.

© 2022 Park AL et al. *JAMA Network Open*.

In the framework of the net benefit approach, the upper limit of the 95% confidence interval represents the maximum willingness- to- pay (MWTP), which is necessary to gain one additional unit of the outcome criterion with a 95% probability. That means that if a decision maker is willing to pay the defined MWTP for the gain of one extra unit of the outcome by a particular intervention, the intervention can be assumed to be cost-effective with a probability of 95%[1].

© 2022 Park AL et al. *JAMA Network Open*.

eReferences

1. Glick H, Doshi JA, Sonnad SS. *Economic evaluation in clinical trials.* 2nd ed. Oxford: Oxford University Press; 2014.
2. Willan AR, Briggs AH. *Statistical analysis of cost-effectiveness data.* Chichester; 2006.
3. Efron B, Tibshirani R. Bootstrap Methods for standard errors, confidence intervals, and other Measures of statistical accuracy. *Statistical Sciences.* 1986;1:54–77.
